# Myristoyl-CoA:protein *N*-myristoyltransferase depletion in trypanosomes causes avirulence and endocytic defects

**DOI:** 10.1016/j.molbiopara.2009.09.006

**Published:** 2010-01

**Authors:** Helen P. Price, M. Lucia S. Güther, Michael A.J. Ferguson, Deborah F. Smith

**Affiliations:** a Centre for Immunology and Infection, Department of Biology/Hull York Medical School, University of York, York YO10 5YW, UK; b Division of Biological Chemistry and Drug Discovery, College of Life Sciences, University of Dundee, Dundee DD1 5EH, UK

**Keywords:** Arf, ADP-ribosylation factor, Arl, ADP-ribosylation factor-like, BSF, bloodstream form, ConA, concanavalin A, NMT, myristoyl-CoA:protein *N*-myristoyltransferase, RNAi, RNA interference, *T. brucei*, *Trypanosoma brucei*, VSG, variant surface glycoprotein, Myristoyl-CoA:protein *N*-myristoyltransferase, *N*-Myristoylation, *Trypanosoma brucei*, Endocytosis, RNA interference, Drug target

## Abstract

The enzyme myristoyl-CoA:protein *N*-myristoyltransferase (NMT) catalyses the co-translational covalent attachment of the fatty acid myristate to the N-terminus of target proteins. NMT is known to be essential for viability in *Trypanosoma brucei* and *Leishmania major*. Here we describe phenotypic analysis of *T. brucei* bloodstream form cells following knockdown of NMT expression by tetracycline-inducible RNA interference. Cell death occurs from 72 h post-induction, with approximately 50% of cells displaying a defect in endocytic uptake by this time. The majority of these induced cells do not have an enlarged flagellar pocket typical of a block in endocytosis but vesicle accumulation around the flagellar pocket indicates a defect in vesicular progression following endocytic fusion. Induced parasites have a wild-type or slightly enlarged Golgi apparatus, unlike the phenotype of cells with reduced expression of a major *N*-myristoylated protein, ARL1. Critically we show that following NMT knockdown, *T. brucei* bloodstream form cells are unable to establish an infection in a mouse model, therefore providing further validation of this enzyme as a target for drug development.

*N*-Myristoylation is required for the function of a range of eukaryotic and viral proteins, including the ADP-ribosylation factors (Arfs), Ras, the Src tyrosine kinase and HIV gag protein [1]. The enzyme catalysing this co-translational process is myristoyl-CoA:protein *N*-myristoyltransferase (NMT). We previously reported that NMT is essential for viability in the protozoan parasites *Leishmania major* (by gene targeting via homologous recombination) and *Trypanosoma brucei* (by RNA interference, RNAi) [2] and is thus a promising target for the development of novel therapeutics [3]. In a proof-of-principle study, two compounds with activity against fungal NMTs were able to inhibit purified *T. brucei* NMT *in vitro* and severely compromise growth of the bloodstream form (BSF) of the parasite in culture [3]. Here, we present more extensive characterisation of the phenotype observed following knockdown of NMT expression in BSF *T. brucei*, both *in vitro* and in an *in vivo* infection model, providing further validation for TbNMT as a putative drug target.

RNAi knockdown in bloodstream form *T. brucei* decreases NMT-specific transcripts to 57% of the original level by 4 h post-induction, as measured by real time PCR (data not shown). No further reduction is seen by 24 h post-induction. Loss of NMT protein occurs by 72 h post-induction, at which time there is a decrease to 16% of the original level. This time correlates with the onset of cell death in both procyclic and bloodstream forms of the parasite [2]. The data presented here reveal no dominant effects on progression through the cell cycle in RNAi treated cells, with only a minor accumulation (<5%) of cells with abnormal kinetoplast: nucleus configurations by 96 h post-induction (Fig. 1A). There is a marked increase in the number of round and tear-shaped cells, peaking at 84 h post-induction (14% and 9%, respectively). However, the majority of cells show no gross morphological abnormalities during the time course studied (Fig. 1B).

The survival of BSF trypanosomes depends on the correct functioning of extremely rapid transport mechanisms, for which at least two *N*-myristoylated proteins, TbARL1 and TbARF1, are known to be required [4,5]. Therefore, specific trafficking events have been analysed in detail in the NMT-depleted cells. *T. brucei* BSF are able to internalise the lectin concanavalin A (ConA) at the flagellar pocket membrane via receptor-mediated endocytosis, trafficking the lectin through the endocytic system before it reaches the terminal lysosome. Following NMT knockdown, the proportion of cells defective in the uptake of ConA increases steadily (Fig. 1C and D) reaching approximately 50% by 72 h post-induction. In contrast, the glycoprotein p67 is clearly detectable in the lysosome, suggesting no gross defect in ER-Golgi-lysosome trafficking (Fig. 1D). The transport of newly synthesised variant surface glycoprotein (VSG) from the ER onto the plasma membrane, measured by its ability to be released in a soluble form by osmotic shock [6,7], appears to be unaffected by NMT depletion (Fig. 1E). However, the total incorporation of radioactivity into RNAi-induced cells as measured by densitometry is 25% of that seen for the control cells (Fig. 1E, data not shown), indicating a decrease in protein synthesis, which could in turn mask a defect in this transport pathway.

Electron microscopy shows that RNAi-induced parasites have a normal or slightly enlarged Golgi apparatus (68% normal size and 32% enlarged, *n* = 50 cells where the Golgi was visible) (Fig. 1G–I) compared to the parental cell line (Fig. 1F). Many cells also exhibited an accumulation of vesicles around the flagellar pocket (Fig. 1J and K). Four or more vesicles were found around the flagellar pocket of 72% of induced cells, compared to less than 10% of cells from the parental line (*n* = 50 cells where the flagellar pocket was visible). This observation suggests that cargo can be internalised but is not being appropriately transported from the flagellar pocket to the endocytic system. Therefore these data support the proposal that loss of NMT affects proteins required for vesicular progression rather than the endocytic fusion event.

Bioinformatic analysis predicts the presence of approximately 60 putative *N*-myristoylated proteins encoded by the *T. brucei* genome [8]. The depletion of NMT is therefore likely to have pleiotropic effects, influencing several divergent pathways and functions. The phenotype presented here differs significantly from that seen following knockdown of the essential *N*-myristoylated small GTPases, TbARF1 and TbARL1 in BSF [4,5]. Loss of each of these causes the appearance of multinucleated cells prior to death, indicative of a defect in cytokinesis followed by unchecked cell cycle progression. Knockdown of TbARL1 in BSF cells results in disintegration of the Golgi apparatus, accumulation of vesicles and multivesicular bodies in the cytosol and an apparent defect in the transport of VSG to the cell surface [4]. Although Arf1 is a key component of the secretory system in other eukaryotes, RNAi of the Arf1 orthologue in *T. brucei* BSF results in the ‘Big Eye’ phenotype. This was first described in cells following knockdown of the clathrin heavy chain [9], in which a block in receptor-mediated endocytosis (but ongoing exocytosis) correlated with the appearance of rounded cells displaying gross enlargement of the flagellar pocket. The Big Eye phenotype is not evident in the majority of NMT-depleted cells, possibly because the defect in uptake in these cells occurs following endocytic fusion.

The effects of NMT depletion have also been tested *in vivo* in a rodent model. Due to the observed lack of virulence of our 90-13 cell lines, it was necessary to produce a new NMT RNAi cell line derived from BSF Lister 427. The *in vitro* growth kinetics of these new parasites post-induction (Fig. 2A) are very similar to the original NMT RNAi cell line [2], with an initial decrease in growth rate compared to uninduced cells, followed by the onset of cell death by 72 h. Following the induction of RNAi for only 24 h, *T. brucei* BSF are severely compromised in their ability to establish an infection in BALB/c mice (Fig. 2). No induced parasites can be detected in the sera of infected mice at any time during the 4-day infection (with the figures plotted in Fig. 2B at the lower limit of detection for undiluted sera, corresponding to the maximum possible parasitaemia in these mice). The molecular mechanism leading to this inability to establish in the mammalian host is unknown, but defects in VSG trafficking are likely to play a major role in this effect.

NMT has been shown to be an essential protein in many eukaryotic cells, including *Saccharomyces cerevisiae* [10], *Cryptococcus neoformans* [11], *Candida albicans* [12], *Caenorhabditis elegans*, *Drosophila* [13] and mammals [14,15]. A gel shift assay was employed to demonstrate loss of *N*-myristoylation of the highly conserved GTPase Arf1 in *C. albicans* following disruption of NMT function by treatment with inhibitory compounds [16]. As Arf1 is a vital component of the secretory pathway and *N*-myristoylation is crucial for the correct localisation of this molecule, this effect alone may be sufficient to cause lethality. However, as approximately 0.5% of eukaryotic proteins have a putative *N*-myristoylation motif [1], the effects of NMT depletion are likely to be significantly more widespread. Null mutation of the single NMT gene in *Drosophila* causes disruption of the actin cytoskeleton and ectopic apoptosis in embryos, possibly attributed to loss of function of myristoylated tyrosine kinases [13], which are absent from the *T. brucei* proteome. Mammalian genomes encode two related but distinct NMT isoforms, NMT1 and NMT2 (77% identity) which are differentially regulated during development. NMT1 is the dominant isoform expressed during early embryogenesis in mice and is essential for viability during this period [14]. Analysis of heterozygous knockout mice revealed that NMT1 is required for monocytic differentiation of bone marrow cells [17]. Knockdown of either of the two human NMT isoforms results in an increase in apoptosis due to altered regulation of the BCL2 family of proteins [15]. In this current study, we have characterised the effects of NMT depletion on the unicellular trypanosome, demonstrating that loss of this protein results in a defect in endocytic uptake, generating parasites that are severely compromised in their ability to infect the mammalian host. As *N*-myristoylation is a critical process in eukaryotes, the identification of highly specific inhibitors will be paramount to the successful development of new therapeutics targeted to the *T. brucei* NMT enzyme.

## Figures and Tables

**Fig. 1:**
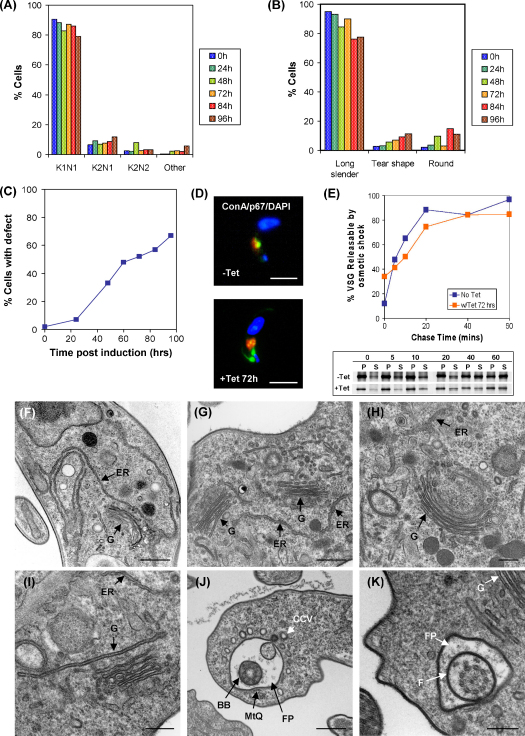
Effects of NMT RNAi on cell morphology and intracellular transport. (A) Nuclei and kinetoplasts were counted in NMT RNAi parasite line Bp2T7/NMT (90-13) [2] grown in the absence or presence of tetracycline over a 96 h time course. At least 250 cells were counted per sample. (B) Abnormal cell morphology was quantified by light microscopy in the BSF line as above over a 96 h time course following addition of tetracycline. The percentage of tear-shaped and round cells are shown for each time point. At least 250 cells were scored per sample. (C) Receptor-mediated endocytosis was analysed by monitoring the uptake of FITC-labeled ConA in BSF line as above grown in the presence of tetracycline for 0–96 h. Cells labeled with FITC-ConA but with no co-localisation with p67 were classified as having an endocytic defect. >100 cells were counted per experimental group. (D) Typical uninduced and tetracycline induced (72 h) cells, the latter showing a defect in ConA uptake. Cells are labeled with FITC-ConA (green), p67 (red) and DAPI (blue). Bar, 5 μm. (E) VSG exocytosis was monitored in BSF line as above, grown in the absence and presence of tetracycline for 72 h. VSG was isolated from lysates of cells labeled by pulse-chase with [^35^S]-labeled methionine and cysteine. Radiolabeled products were separated by SDS-PAGE, detected by autoradiography and quantified by densitometry. Results are presented as the % VSG trafficked from the endomembrane (insoluble) fraction to the plasma membrane (soluble) fraction. Data shown are representative of two independent experiments. The lower panel shows the autoradiograms from one experiment, exposed for the same length of time. P, pellet; S, soluble. Electron micrographs of parental BSF (F) and the RNAi line as above (G–K) grown in the presence of tetracycline for 72 h. ER, endoplasmic reticulum; G, Golgi apparatus; CCV, clathrin-coated vesicle; BB, basal body; MtQ, FAZ-associated microtubule quartet; FP, flagellar pocket; F, flagellum. Bar, 0.5 μm (F, G, and J) or 0.25 μm (H, I, and K).

**Fig. 2:**
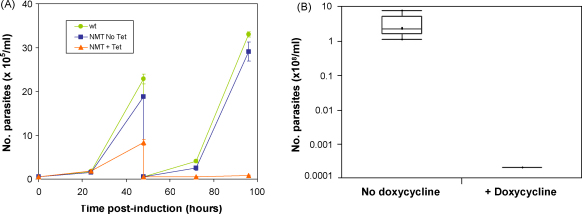
Effects of NMT RNAi on infectivity. (A) Growth of the parental line (wt) and NMT RNAi parasite line Bp2T7/NMT (Lister 427) grown in the presence and absence of tetracycline monitored over a 5-day time course. (B) Parasitaemia in BALB/c mice 4 days following infection with 5 × 10^5^ parasites of the NMT RNAi line as above. Parasitaemia was measured by microscopic analysis of tail-cut blood samples. Parasites were grown in the absence or presence of tetracycline for 24 h prior to infection. Mice given tetracycline-treated cells were given doxycycline in drinking water from 1 week prior to infection. *n* = 5 mice for −dox and for +dox. No parasites were detected in any of the mice infected with the +dox cell line. The value shown for this group is the lower limit of detection (1E4/ml), the maximum possible parasitaemia for these mice.
